# Adaptive changes in sensorimotor processing in patients with acute low back pain

**DOI:** 10.1038/s41598-022-26174-2

**Published:** 2022-12-16

**Authors:** Bart Boendermaker, Roman Buechler, Lars Michels, Jo Nijs, Iris Coppieters, Sabina Hotz-Boendermaker

**Affiliations:** 1 Department of Neuroradiology, University Hospital Zurich, Zurich, Switzerland; 2 Pain in Motion Research Group, Department of Human Physiology and Physiotherapy, Faculty of Physical Education & Physiotherapy, Vrije Universiteit Brussel, Brussels, Belgium; 3 Department of Physical Medicine and Physiotherapy, University Hospital Brussels, Brussels, Belgium; 4 Neuroscience Center Zurich, University of Zurich and Swiss Federal Institute of Technology Zurich, Zurich, Switzerland; 5 School of Health Professions, Institute of Physiotherapy, University of Applied Sciences ZHAW, Winterthur, Switzerland

**Keywords:** Health care, Rheumatology, Pain, Chronic pain

## Abstract

In low back pain (LBP), primary care and secondary prevention of recurrent and persistent LBP are not always successful. Enhanced understanding of neural mechanisms of sensorimotor processing and pain modulation in patients with acute LBP is mandatory. This explorative fMRI study investigated sensorimotor processing due to mechanosensory stimulation of the lumbar spine. We studied 19 adult patients with acute LBP (< 4 weeks of an acute episode) and 23 healthy controls. On a numeric rating scale, patients reported moderate mean pain intensity of 4.5 out of 10, while LBP-associated disability indicated mild mean disability. The event-related fMRI analysis yielded no between-group differences. However, the computation of functional connectivity resulted in adaptive changes in networks involved in sensorimotor processing in the patient group: Connectivity strength was decreased in the salience and cerebellar networks but increased in the limbic and parahippocampal networks. Timewise, these results indicate that early connectivity changes might reflect adaptive physiological processes in an episode of acute LBP. These findings raise intriguing questions regarding their role in pain persistence and recurrences of LBP, particularly concerning the multiple consequences of acute LBP pain. Advanced understanding of neural mechanisms of processing non-painful mechanosensations in LBP may also improve therapeutic approaches.

## Introduction

Low Back Pain (LBP) is a global public health concern that poses substantial medical and economic challenges^1,2^. Until recently, there has been consensus that acute LBP is a self-limiting condition, with a minority of patients developing persistent pain^3^. However, this assumption relates mainly to cross-sectional studies^2^. Strong evidence indicates that two-thirds of patients still report low to moderate pain from three to 12 months^4,5^, and many patients will experience a relapse within a year^6^.

The cause for persistent LBP is yet to be determined and is not merely a result of peripheral nociceptive input. Instead, neuroscientific research revealed nervous system adaptations regarding central pain processing that mediate and maintain LBP^7,8^. In the (sub)acute phase, neuroplastic changes are a natural, adaptive biological process influenced by biophysical, psychological, and social factors^9^. In the chronic stage, they become maladaptive^7,10^. Neuroimaging is increasingly used to evaluate these so-called maladaptive changes^7,10^.

Regarding brain function, the opinion prevails that brain processes engage large-scale networks where brain regions continuously exchange information rather than isolated locally activated or deactivated areas^11,12^. Neural associations form distributed large-scale reciprocal connections that transiently link local networks^13^. Large-scale interactions imply that the activity of regions in the network demonstrates strong correlations with the activation of another remote area within the network. Thus, investigations targeting connectivity between specific brain regions and local networks involved in sensorimotor processing may provide insights into the brain organization and communication in patients with LBP while capturing the multidimensional facets of pain^14,15^. One such critical large-scale network is the salience network (SN), which attends and integrates afferent sensory input and recruits additional functional networks involved in emotion, reward, or motivation to modulate behavior^16–18^. A refined understanding of the interaction between these networks involved in sensory processing in LBP patients may effectively target interventions^19,20^. Recent work has validated the presence of somatotopic-specific alterations in resting-state functional connectivity, finding increased connectivity between the nociceptive representation of the back and the salience network in patients with LBP^15^. Similarly, a systematic review on cortical reorganization in persistent LBP reported alteration in functional connectivity and increased activity following painful stimulation^7^.

The evidence in the literature mainly originates from patients suffering from persistent LBP and using painful stimulation. There is a need to clarify network dynamics in the acute LBP episode, thus within the first four weeks after the onset of pain^21^. This point is striking as knowing an early treatment approach may prevent persistent or recurrent pain. Chang and colleagues reported lower sensorimotor activity in somatosensory cortices due to non-painful stimulation in acute LBP patients (< 4 weeks)^22^. Individual variations in processing non-noxious stimulation in the secondary somatosensory cortex (S2) and anterior cingulate cortex (ACC) were detected^22^.

We have developed a functional magnetic resonance imaging (fMRI) paradigm to investigate neural correlates of sensorimotor functioning of the lumbar spine and defined the cortical sensorimotor representation and somatotopy of the lumbar spine in healthy persons^23,24^. The resulting activation pattern has been considered a proxy for anticipatory postural control and a promising area to investigate the effects of LBP on the sensorimotor system. In patients with persistent LBP, a cross-sectional fMRI study from our group reported a reorganization of higher-order processing for sensory information. At the same time, the primary somatosensory cortex (S1) representation remained unchanged^24^.

The main aim of this investigation was to use fMRI to explore non-painful sensorimotor processing in patients with acute LBP. Task-related activation analysis should reveal the regions specifically activated during somatosensory processing in patient and control groups. Then, task-related functional connectivity analyses explored large-scale networks of regions strongly interacting during this task. The objective of this study was to shed light on the time-point of adaptive changes in acute LBP; no study has investigated this issue before. Hence, the present study addressed a critical knowledge gap. We hypothesized that our findings should enhance our understanding of neural mechanisms of sensorimotor processing and influence factors in pain modulation in patients with acute LBP. Furthermore, results should enhance our understanding of neural mechanisms of sensorimotor processing and influencing factors in pain modulation in patients with acute LBP. Eventually, the findings could help increase the success of primary care and secondary prevention of recurrent and persistent LBP.

## Results

### Clinical and psychometric data

The baseline characteristics of the patient group are displayed in Table 1. The patients with acute LBP reported moderate mean pain intensity of 4.5 on a numeric rating scale (NRS). In individual patients, it ranged from mild (2/10) to severe (8/10). Three of the 17 patients with LBP took non-steroidal anti-inflammatory drugs (NSAIDs) for pain treatment. The LBP-associated disability measured with the Oswestry Disability Index indicated a mild mean disability of < 20%, and the range was not extending a moderate level of < 40%. Similarly, the group mean of the patients did not indicate mental health problems. There were few indications of depressive mood (N = 5) and stress symptoms (N = 5), but none of anxiety, as measured with the Depression, Anxiety, and Stress Scale. In contrast, the group's mean state anxiety score (42.9) indicated moderate anxiety, ranging from moderate to high. State anxiety reflected the stressful condition of acute pain and indicated the fleeing emotional state of apprehension and nervousness.

**Table 1:** Patients Baseline Characteristics (n = 17).

Questionnaires and clinical Data	Mean (SD); range or %
*Pain intensity (Numeric Rating Scale)*	4.5 (1.2); 2–8
*Disability (Oswestry Disability Index)*	12.8 (6.7); 0–31
*Depression anxiety stress scale*	
Depression	4.5 (4.6); 0–14
Anxiety	1.9 (2.6); 0–8
Stress	6.9 (5.2); 0–16
*State anxiety index*	42.9 (4.3); 35–54
*Pain responses (Avoidance Endurance Questionnaire)*	
Avoidance of physical activities	3.1 (1.6); 0.4–6
Pain persistence scale	3.1 (0.8); 2–5
*Previous episodes of low back pain*	
None	12%
1–2	29%
3–4	12%
> 4	47%
*Mean number of days sick leave*	1.81 (3.8); 0–28
**Clinical assessments**	
*Movement control impairment test*	2.3 (1.2); 0–6
*Two-point discrimination vertical in cm*	
Right	3.7 (1.3); 2–7.5
Left	3.8 (1.6); 2–8.6
*Pressure Pain Threshold*	
Right	637.9 (366.8); 211–1694
Left	655.6 (343,4); 254–1616
*Wind up ratio (Pinprick)*	
Right	1.5 (0.6); 0.8–3
Left	1.7 (0.9); 0.5–3.5

### Clinical assessments

The clinical assessment consisted of tests for spinal movement control, two-point discrimination for tactile acuity, pressure pain threshold for tissue sensitivity, and wind-up ratio as a surrogate for central sensitization. The outcomes of these tests are reported in Table 1. The movement control test battery revealed a mean of two positive (i.e., inaccurately performed) tests and a range from zero to six. There is evidence of impaired movement control by at least two positive tests in N = 9 participants. The mean two-point discrimination of 3.7 resp. 3.8 cm is comparable to previous reports in healthy subjects^25^. Finally, the wind-up ratio revealed a substantial deviation, suggesting that some patients already showed features of central sensitization in the acute phase of LBP.

### Event-related functional MRI analysis

Lumbar spine stimulation resulted in a bilaterally hemodynamic response in the control and patient group in parietal and central opercular cortices (S2). In addition, in the LBP group, but not in the healthy control group, we found activation in the central region (i.e., the postcentral and precentral gyrus) and the anterior cingulate gyrus in the right hemisphere (Supplementary Information, Table 1). Furthermore, considerable clusters were found in the right postcentral gyrus groups in the control group and bilaterally in the juxtapositional lobule (supplementary motor area, SMA). These foci of the activation did not survive cluster-correction threshold. The contrast between the patient group and the controls revealed a cluster in the paracingulate gyrus (MNI coordinates: -6, 36, 26, Z = 3.38, voxel size = 34, *P* > 0.001) did not reach a significance level. The control group did not display enhanced activation compared to the patient group.

### Functional connectivity analysis using a general psychophysiological Interaction (gPPI)

To identify brain regions that demonstrated mechanosensory-related differences in functional connectivity, we ran separate gPPI analyses for large-scale brain networks, including the salience, limbic-parahippocampal, cerebellar, and sensorimotor networks. Specifically, the analysis computed the interaction term reflecting the modulation of the connectivity strength when changing from rest to stimulation condition. The result section reports the interaction term group differences between healthy subjects and acute LBP patients. Notably, there was an apparent divide in the modulation of the connectivity strength for the different groups. Decreasing connectivity was found in the patient group’s salience and posterior cerebellar networks, while connectivity values in healthy controls increased. In contrast, the limbic-parahippocampal networks revealed an enhancement of connectivity in the patient group and a significant decrease in healthy controls. No significant differences were observed for lateral and superior sensorimotor networks.

**Figure 1:**
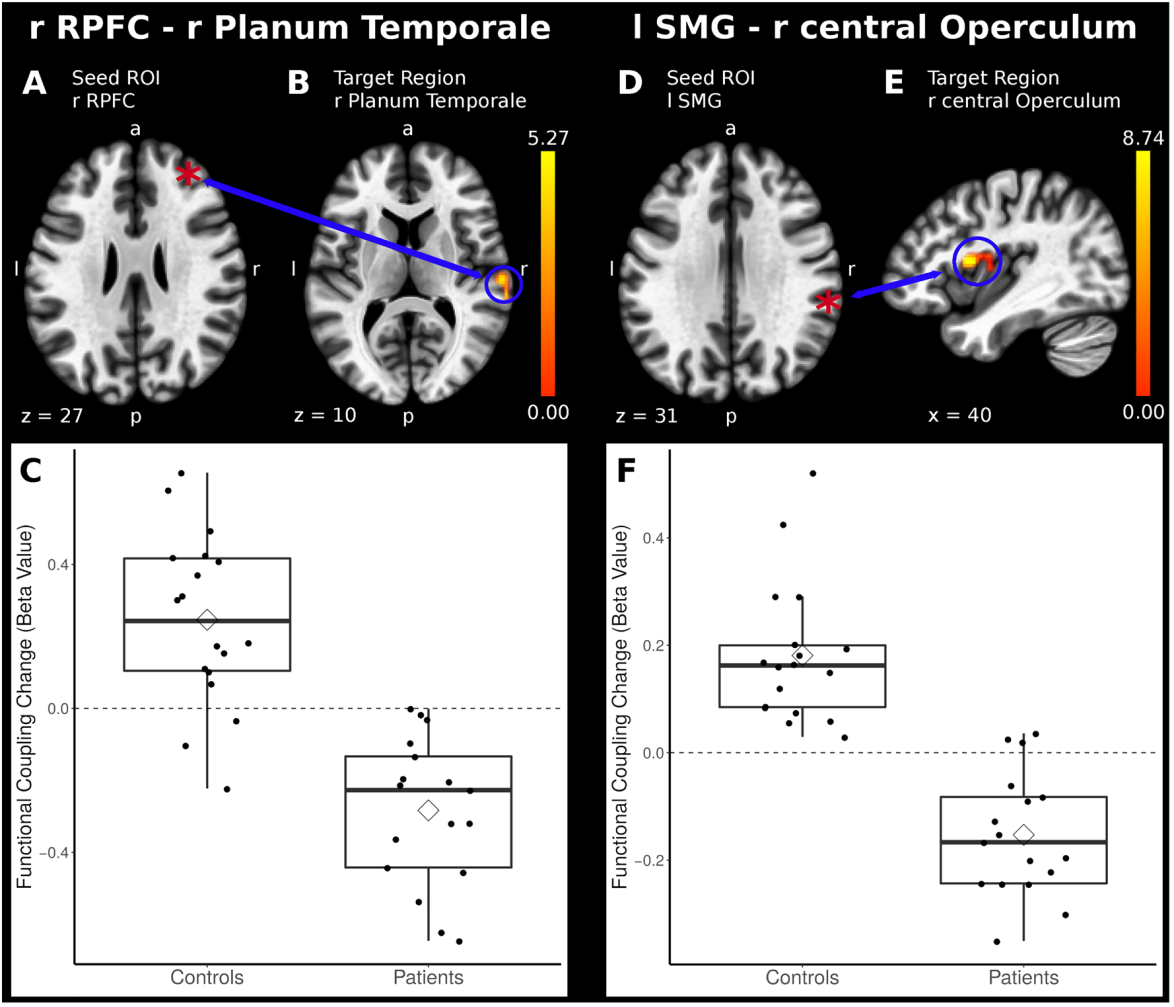
Within the salience network, the seeds *rostral prefrontal cortex* and *supramarginal gyrus (SMG)* demonstrated a decrease in mechanosensory-related functional connectivity with the temporal and opercular region as a function of behavioral adaptation (*P* < 0.05 FWE-corrected, the coordinates are displayed in Table 2). Below are the boxplots of the Beta Values for the patients and control group.

Using seed ROIs of the salience network, significant differences in connectivity strength modulation were detected, i.e., connectivity in patients decreased during stimulation vs. rest while it increased in healthy controls. This pattern was observed between the left supramarginal gyrus (SMG, seed ROI) and the central operculum (anterior insula/S2, Table 2, Fig. 1) in the right hemisphere and between the rostral prefrontal cortex (seed ROI) and the planum temporale and the parietal resp. central operculum (insula/S2, Fig. 1). In addition, a decrease of connectivity in patients vs. an increase in controls was found between the right paracingulate gyrus and the postcentral gyrus as well as between the right anterior insula and the left supramarginal gyrus. However, these additional results did not survive the correction for multiple comparisons.

**Table 2:** Displayed are differences between patients with low back pain and a control group for locations of task-related functional connectivity of non-painful posterior to anterior intervertebral movement of the lumbar spine in long-ranging networks involved in sensorimotor processing.

Networks	Seed	Voxel	p(FDR)	R/L	x	y	z	Brain regions	Functional region	Beta value	T value
**MNI peak coordinates**											
*Salience*	Supramarginal Gyrus_L	238	** < *0.000***	R	40	10	14	Central Opercular Cortex/Insular Cortex	Insula/S2	0.33	8.03
	Rostral prefrontal cortex_R	300	** < 0.000**	R	64	-20	10	Planum temporale: Parietal/Central Operculum Cortex/Insular Cortex	Insula/S2	0.53	7.01
	Paracingulate Gyrus_R	40	> 0.05 uncorr	R	34	-28	42	Postcentral Gyrus	S1/S2	0.54	5.4
		36	> 0.05 uncorr	R	64	-18	30	Postcentral Gyrus	S1/S2	0.7	4.68
	Anterior Insula_R	33	0.05 uncorr	L	-64	-48	16	Supramarginal Gyrus, posterior division		0.37	4.71
	Cingulate cortex, anterior division	65	> 0.05 uncorr	L	-22	16	52	Superior Frontal Gyrus		-0.73	-5.02
*Limbic and anterior Parahippo campal*	Hippocampus_R	129	**0.03**	R	6	-76	-26	Cerebellum Crus I R		-0.89	-5.21
		105	**0.04**	R	34	-68	-32	Cerebellum Crus I R		-0.58	-5.94
		91	**0.04**	R	42	-***62***	34	Lateral occipital cortex, superior division R		-0.65	-4.90
	Hippocampus_L	191	**0.003**	R	38	-58	-26	Cerebellum Crus I R		-0.55	-6.35
	Amygdala_R	133	> 0.05 uncorr	R	6	50	10	Paracingulate Gyrus/Precentral R		0.42	4.72
		101		R	10	46	20	Paracingulate Gyrus		0.3	4.84
		43		R	36	-20	58	Precentral R		0.27	4.76
	Amygdala_L	44	> 0.05 uncorr	L	-42	-2	-8	Insular Cortex L	Insula	0.57	4.49
*Cerebellar_posterior*		303	** < *0.000***	L	-8	6	62	Juxtapositional Lobule Cortex	SMA	0.4	6.38
								Superior Frontal Gyrus			
*Sensorimotor*	Superior	43	> 0.05 uncorr	R	4	30	-10	Cingulate Gyrus anterior division		0.91	4.88
	Lateral_R	35	> 0.05 uncorr	R	28	48	24	Frontal Pole		0.65	4.84
	Lateral_L	35	> 0.05 uncorr	R	34	-96	2	Occipital Pole		0.47	5.63

**Figure 2:**
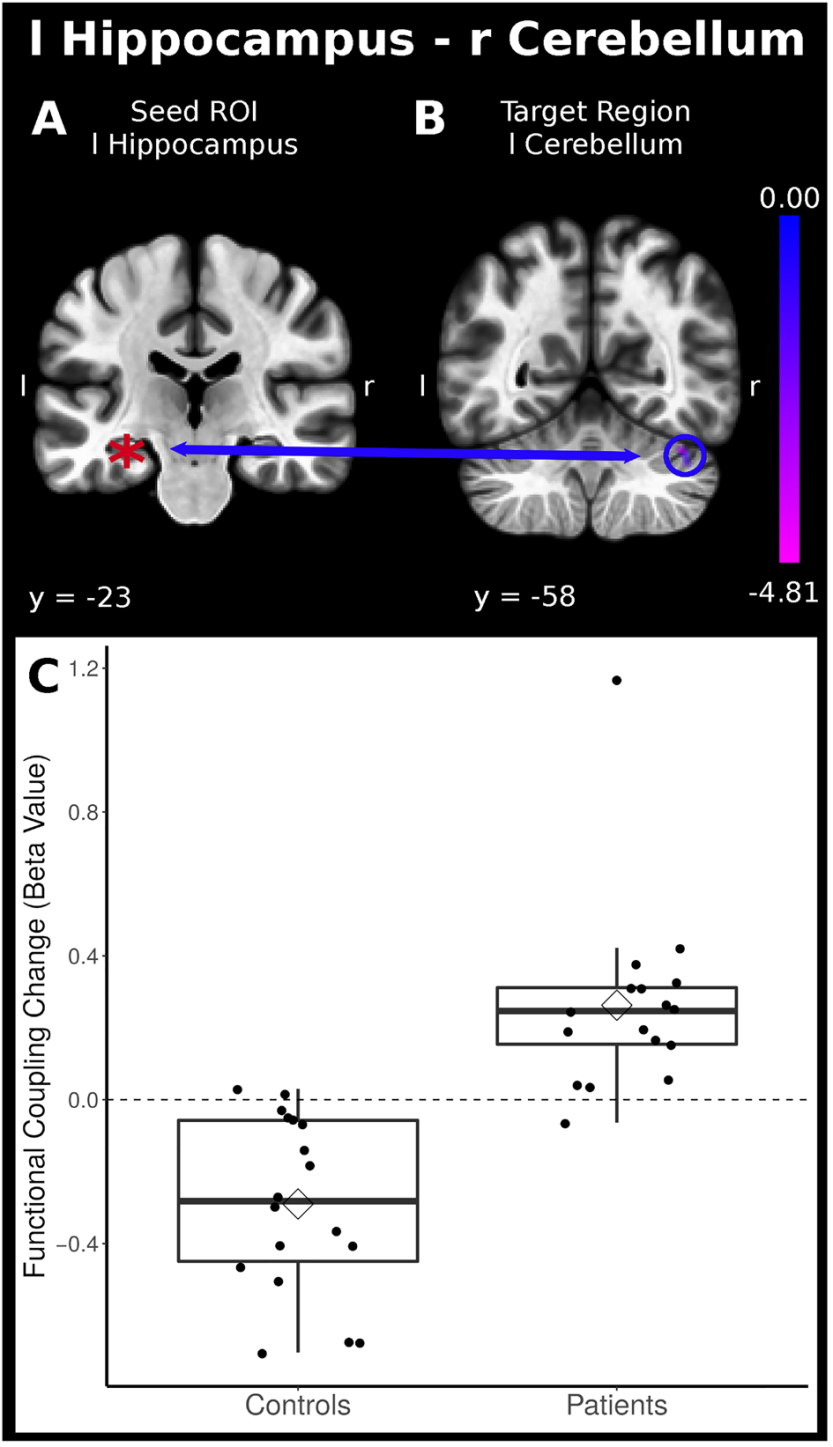
For the limbic and anterior parahippocampal network, increased task-related functional connectivity was disclosed between the seed *hippocampal gyrus* and the right cerebellum as a function of behavioral adaptation (*P* < 0.05 FWE-corrected, the coordinates are displayed in Table 2). Below are the boxplots of the Beta Values for the patients and control group.

In the limbic-parahippocampal networks, the gPPI analysis revealed an enhancement of connectivity strength in patients and a reduction in controls bilaterally between the hippocampus and the right cerebellum crus 1 and 2 (Table 2, Fig. 2). In addition, during stimulation, the right hippocampus showed increased connectivity with the right angular gyrus and cerebellum, vermis 7, while in the controls, the strength decreased. Although uncorrected, attenuation of correlations was detected between the amygdala and paracingulate and precentral cortices in the right hemisphere, and strengthening associations were found between the amygdala and the insular cortex in the left hemisphere in patients when compared to controls.

**Figure 3:**
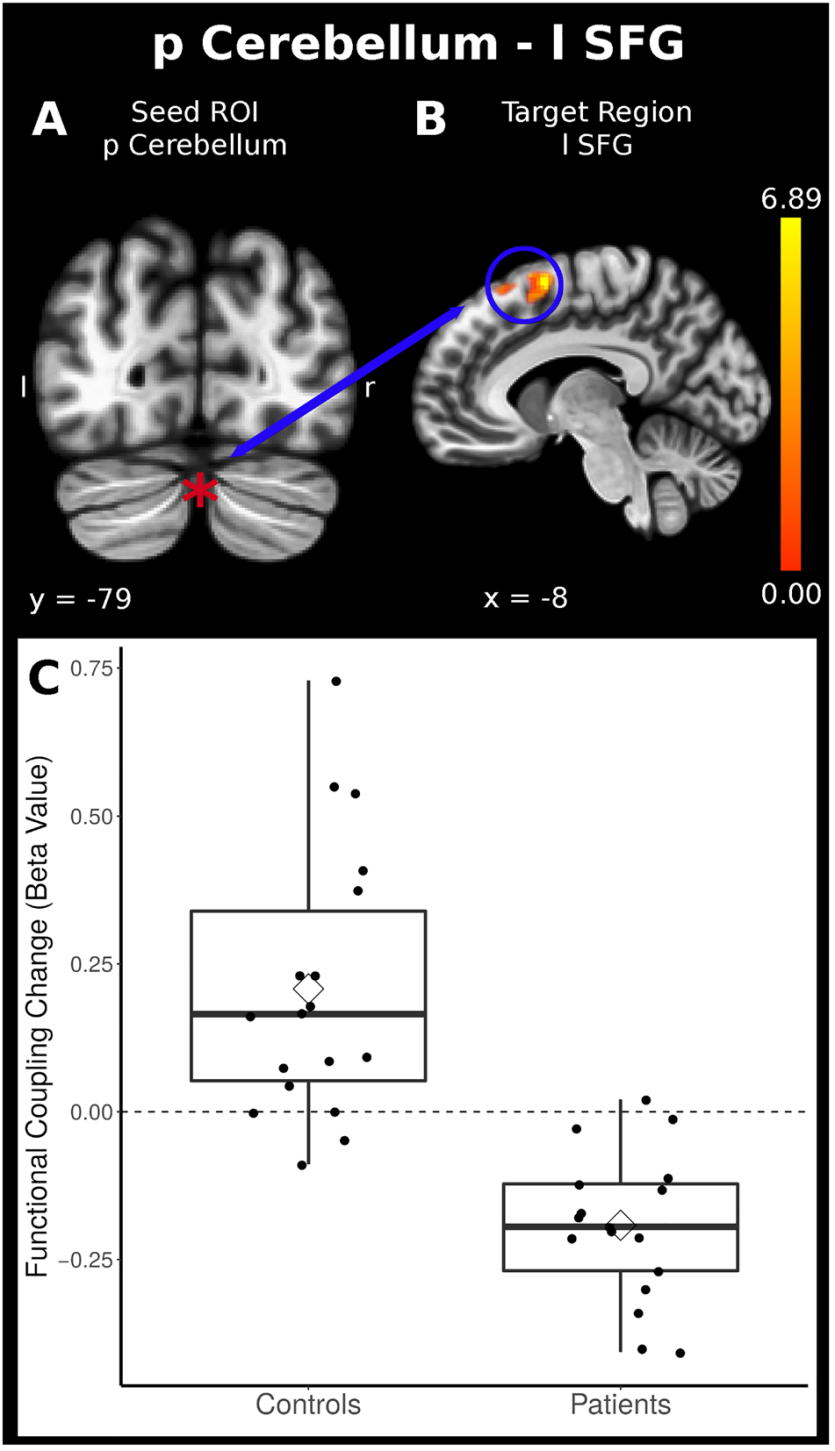
Task-related connectivity was reduced between the *posterior cerebellar network* as the seed region and the left superior frontal cortex (*P* < 0.05 FWE-corrected, the coordinates are displayed in Table 2). Below are the boxplots of the Beta Values for the patients and control group.

Furthermore, in the patients with acute LBP, the posterior cerebellar network revealed reduced connectivity during stimulation with the left superior frontal cortex compared to enhanced connectivity in the control group (Table 2, Fig. 3). Finally, patients with acute LBP displayed weaker connectivity between the posterior cerebellar network and the juxtapositional lobule cortex (SMA), extending to the superior frontal cortex in the midline. This association was stronger in controls.

The sensorimotor networks comprise the pre- and postcentral gyri. In patients with LBP, decreased connectivity was revealed in the right hemisphere between the superior portion of the network and the anterior division of the cingulate gyrus. A decrease in connectivity strength was found for the seed lateral sensorimotor network on the right side with the frontal pole and in the left hemisphere with the occipital pole. However, after correcting for multiple comparisons, these differences in sensorimotor networks remained insignificant.

## Discussion

This explorative fMRI study was the first to investigate functional changes within four weeks of an acute LBP episode. The event-related fMRI analysis yielded no differences between patients with acute LBP and healthy controls. However, the computation of gPPI revealed differences between the patient and control group in a priory-defined networks involved in sensorimotor processing. In the patient group, decreased connectivity was detected in the salience and cerebellar networks, while enhanced connectivity strength was identified in the limbic and parahippocampal networks. These results support the hypothesis that early changes in connectivity might be an adaptive physiological process in patients with acute LBP. On the other hand, these findings raise intriguing questions regarding their role in pain persistence and recurrences of LBP.

### FSU movements, a proxy for anticipatory postural control

The paradigm investigated cortical processing of mechanosensory input involved with trunk movement control. Sensory information is mandatory to select neural strategies for anticipatory postural adjustments to counteract forthcoming perturbation^26^. We manually mobilized FSU’s to simulate lumbar spine movements that will trigger anticipatory postural adjustments^23^. As the spine’s musculoskeletal system is dense with mechanosensors and proprioceptors, motions induce substantial afferent input^27^. Task-related activation patterns revealed robust activations in both groups in S2 bilaterally and smaller in S1. Although not significant, activation in premotor areas (e.g., SMA) points to subliminally anticipatory motor preparation. The analysis of between-group differences did not yield alterations. Although we previously reported reorganization in a chronic patient group^24^, the time since pain onset might have been too short, prompting reorganization. These findings could indicate that motor control in anticipatory postural adjustments in the acute LBP episodes remained unaffected.

We then performed a general psycho-physiological interaction analysis to consider task-specific communication between brain regions involved in sensorimotor processing. This approach has two advantages compared to the event-related analysis. First, adaptive changes in functional networks occur faster than neural changes in regional brain activation^28^. Secondly, this approach investigated large-scale distributed networks critical to sensorimotor and cognitive processing of afferent mechanosensory information beyond pain perception. The following part describes the functioning of such large-scale networks in acute LBP.

### Salience network

The spine’s afferent mechanosensory information is filtered and amplified at multiple levels in neural pathways^18^. Ultimately, the brain’s salience network (SN) decides on a stimulus’s prominence, deviation, or emotional content relative to the surrounding noise^15,17^. Hence, within the “responsibility” of the SN lies the suppression of afferent sensory information irrelevant to a given task^19,29^. In the SN, the patients showed decreased associations between the left SMG and the right central operculum/insula and between the right prefrontal cortex and the right planum temporale during stimulation. The controls showed an enhancement in connectivity. The posterior insula/S2, located in the opercular region, forms a central part of the SN. It links sensory information with essential body functions such as homeostatic, cognitive, and affective systems and mediates interoceptive awareness and physiological reactivity^17,19^. The SMG’s main task lies in the higher-order processing of proprioception and tactile information and the perception of body location in space^30^. This information is mandatory for coordinating and motor preparation for trunk movements^31^. Therefore, it is puzzling that the flow of information from the spinal stimulation seemed less timed and conceivably less salient in the patient group, as revealed by the decreased connectivity. Similarly, we detected decreasing connectivity in patients between prefrontal areas and the planum temporale/S2 region while connectivity in controls increased. Typically, within the salience network, these cortical areas process sensory signals as a part of the sensory working memory. Thus, the salience network approaches the sensory working memory for appraising ongoing sensory afferents^32^. The retained information enables a match with memorized information, stored as a pattern for physiological motor control. Subsequently, there is a transformation in forthcoming behavior^33^. Neuroimaging evidence has revealed increased activity in these brain areas during sensory memory tasks^33^.

What could be the consequences of the described decreases in processing non-painful information? We hypothesize that our results might indicate the SN’s critical function to detect and react to nociceptive input in LBP, noxious by nature, that during trunk movements flood the system and neglect non-painful mechanosensory information^34^. In controls, the stimuli might initiate an evaluation of the mechanosensory information suppressed in patients due to their expectations of pain. The system’s motivation for behavior adaptation could be a purposeful strategy to protect the lumbar spine from further tissue damage at the expense of processing non-painful mechanosensation^35^. Continuous nociception, perceived as LBP, is a potent stimulus for sensorimotor control changes. Among them, adaptive movement patterns are described as co-contraction, muscular defense tension, or avoiding trunk movement altogether^35,36^. Contemporary views imply that learning processes play a role in adapting sensorimotor control to LBP^37^. So far, the discussion has focused on the appraisal of sensory afferents within the SN. The following section will discuss the orchestration of the adapted behavior.

### Limbic and Hippocampal networks

The hippocampal network and cerebellum contain circuits coordinating sensorimotor learning by timing their outputs together^38^. The cerebellum provides storage capacities and crucial mechanosensory information for motor adaptation and associative learning^39^. The cerebellar function is necessary for motor adaptation, active proprioception, performance modification, and associative learning^40^. Furthermore, the cerebellum pairs the sensorimotor system with cognitive processes by adding crucial mechanosensory information^41,42^. In patients with acute LBP, we found increased connectivity between the hippocampus and the cerebellum (Crus I) within the limbic and hippocampal networks. The enhanced association could reflect the consolidation of a learning process or pain memory, e.g., a conditioned connection, and promote adaptive behavior^43,44^. In particular, the cerebellum influences the formation of habits^45^. Consolidation requires orchestration beyond memory because encoding success depends on various factors in distributed brain networks^12^. A facilitator within this process is motivational content. In particular protective motor control mechanisms that prevent pain experience will be perceived as rewarding^46^. The hippocampus could then play an essential role in reinforcing memories by linking sensory information and inducing adaptation of trunk movement control^47,48^. Interestingly, a closed co-working exists between the hippocampus and prefrontal cortices, thus directly linked to the SN^48^.

In addition, pain-related distress influences sensory processing^49^. The trend for enhanced stimulation-related associations between the amygdala with insula/S2 and the paracingulate gyrus supports this assumption. The amygdala’s primary function is to evaluate emotions and pain, thus deciphering threats within the limbic network^43^. Moreover, the amygdala represents the neural correlate of fear of movement, a cognitive construct widely connected with acute LBP^50^. Thus, long-ranging networks that connect affective and emotional content with motor preparation regions will be outlined next.

### Posterior cerebellar networks

Trunk motor control has been closely associated with the cerebellum and SMA^51^. More recent data described functional connectivity between trunk muscle representation in SMA and cerebellum^52^. Our findings of decreased functional connectivity between these brain areas provide further evidence for adaptive changes within the posterior cerebellar network, potentially affecting trunk motor control. The SMA is central to planning and execution of voluntary motor activities^53^: the timing, sequencing, and amplitude of anticipatory postural muscle activity before actual movement execution^26,54^. The cerebellum integrates the kinematic state of the body to monitor ongoing movements^41^. Interestingly, the cerebellum’s multimodal sensory processing also includes nociceptive information. The cerebellum has the best requisites to influence pain processing, as it receives descending information from other brain areas and ascending nociceptive input^55^. The pain-related adaptations in motor control in acute LBP could thus also influence non-painful sensory processing involved in motor control^56^.

### Sensorimotor networks

Neither the event-related nor the gPPI analysis yielded adaptive changes in S1, while we disclosed S2 alterations in long-ranging networks. This finding was supported by a recent investigation that reported decreased sensorimotor processing in S2 patients with acute LBP^22,57^. Besides, the authors noted that the neuroplastic changes were predictive of pain persistence after six months^57^. Our previous investigation in patients with persistent LBP also showed unaffected S1 organization. An explanation might be the identical representations for nociceptive and non-nociceptive somatosensory input in S1^34^. Besides, a central task of S1 is a dynamic update and evaluation of afferent sensory input. Thus, S1 cannot act as a long-term working memory storage^58^.

### Limitations

All that said, it remains to be discussed how our findings may rely on acute LBP patients. We investigated a pooled group of patients concerning previous LBP episodes. Some participants experienced first-time LBP, while the majority experienced recurrent episodes, which might be considered a different group within the population. Methodological considerations relate to estimating our sample size from the previous studies^23^. We recognize that our estimation may be relatively small and, therefore, only substantial effects are detectable due to the low statistical power. We acknowledge this limitation and suggest that the exciting findings must be interpreted cautiously. Most important, space limitations prevented us from the presentation of correlations with our functional connectivity findings and their relation to clinical variables.

### Conclusion

The present exploratory fMRI study revealed adaptive changes in large-scale networks throughout the brain in patients with acute LBP. These results address a critical knowledge gap and deepen our understanding of whole-brain neurophysiological mechanisms in acute LBP. Besides, they may explain the multiple consequences of acute LBP, particularly concerning anticipatory postural control. Timewise, adaptive changes may occur within the first four weeks of an acute LBP episode. Whether these changes return to normal after the pain subsides is unclear. Further investigation in a patient group in a longitudinal setting may shed light on the time course regarding the sensorimotor alterations and their relation to persistent LBP. Going forward should improve our understanding of the neural mechanisms of processing non-painful mechanosensations in LBP and its impact on behavior. Besides, increasing knowledge may also improve therapeutic approaches.

## Materials and methods

### Subjects

Forty-two participants were enrolled in the study to investigate the somatosensory processing of non-painful stimulation of the lumbar spine. FMRI data from five healthy subjects and two patients were removed due to extensive movement artifacts (2 controls, 1 patient), technical issues concerning the sensors (3 controls), or epilepsy (1 patient). Hence, 18 healthy subjects (8 female, mean age of 31.22 + /- 9.7 years) and 17 patients with acute LBP (9 female, mean age of 31.71, + /- 11.9 years, range 18–60) were included in the final data analysis. Groups were age (two-sample *t*-test, *P* = 0.8955) and sex-matched (Chi-square test, *P* = 0.5799). The sample size was based on previous investigations^23,24^ and empirical investigation that demonstrated that after 20 subjects, the similarity between activation maps would not be significantly improved by adding more subjects^59^. Similarly, Desmond et al. determined that *n* = 24 participants were required to give an accurate activation map with a sufficient power level (i.e., an 80% true positive rate)^60^.

Patients were recruited from physiotherapy practices, two hospital outpatient departments, and a University Campus (UZH) using word of mouth, advertisements, and mailing lists. The sample consisted of a sub-study from a longitudinal observational cohort study on LBP, and these results will be presented elsewhere. A short telephone interview verified the inclusion and exclusion criteria. Patients were included if they experienced LBP for less than four weeks, as acute LBP has been defined as the duration of an episode for less than six weeks^3^. Participants experienced LBP for the first time or in a recurrent episode; however, the patients had to be free of pain for the last six months. The pain was localized between the lower rib and above the inferior gluteal folds, with or without leg pain. Exclusion criteria were a clinically relevant anatomical anomaly in the lower back (e.g. fracture, carcinoma), spine surgery, peripheral or central neurological illness, major psychiatric disorder, pregnancy, or any factor precluding participation in MR imaging. In addition, agents known to affect brain function were excluded. The study was approved by the Canton of Zürich’s Ethics Committee, Switzerland (BASEC-No. 2016–02,096) and was conducted in compliance with the declaration of Helsinki; thus, informed consent was obtained from all subjects**.** Participants were compensated for travel and the burden of participation.

### Experimental procedure

Patients underwent three assessments within seven days, between weeks two to four, after the onset of LBP. First, they had to complete an online survey assessing demographic, clinical, and psychometric data. Then a clinical assessment was completed in a physiotherapy praxis, followed by the MRI acquisition at the University Hospital Zurich. The control group only completed the online survey.

#### An online survey assessing demographic, clinical, and psychometric data

Questionnaire data was collected using an online survey (Survey Monkey). A link was sent by e-mail, and the patients were asked to complete the questionnaires. Patients who did not complete the survey within two days were electronically reminded, followed by a phone call in case of no response.

Information about pain intensity, duration, and location was collected using the painDETECT questionnaire^61^. The mean pain intensity was calculated from the past two weeks' present, worst, and average pain intensity, using a numeric self-rating scale (NRS) ranging from 0 to 10. The Oswestry Disability Questionnaire assessed functional disability due to LBP^62^. The Depression, Anxiety, and Stress Scales screened depression, anxiety, and stress on individual subscales^63^. In addition, we applied the State Anxiety Scale to measure the fleeing emotional state of apprehension and nervousness^64^. The Avoidance-Endurance-questionnaire measured fear-avoidance and endurance-related responses to pain^65^.

#### Clinical assessment

Physiotherapists performed clinical examinations. All investigators underwent a 2-h training session to ensure the intra- and inter-tester reliability of the clinical tests and study procedure. Movement control of the lower back was quantified by applying the Movement Control Impairment test. The test battery assessed six movements and demonstrated a significant difference between patients with LBP and pain-free healthy subjects^66^. Tactile acuity was investigated by applying the vertical two-point discrimination tests performed bilaterally, and the horizontal line, the level of L4, was used as a reference^67^. A dolorimeter quantified the bilateral Pressure Pain Threshold to investigate tissue sensitivity at the same position. The mean pressure pain threshold was computed from three measures with an interval of 30 s between the measurements. Finally, using a toothpick, the temporal summation of pain was tested by applying pinpricks bilaterally at the L4 level. Participants rated pain intensity on an NRS, after two and ten stimulations, with a one-sec interval between stimuli. In the end, the wind-up ratio was determined as the ratio of the stimulus series (10 x) pain intensity / the pain intensities of the individual stimuli (2 x).

### Neuroimaging data acquisition and analysis

#### Lumbar spine paradigm

All participants were scanned in the prone position. The vertebral stimulation consists of non-painful posterior to anterior intervertebral movement (PA) onto the lumbar spinous processes L1 and L4 by the examiner’s thumb^23,24^. The impulse induced a small physiological motion in a functional spinal unit (FSU), representing the same biomechanical features as the entire spine. Anatomically, FSU consists of two adjacent vertebrae, the facet joints, the intervertebral disc, and all adjoining ligaments^68^. The flexible structures are densely packed with mechanosensors and nociceptors, which are mandatory for the spinal control of posture and movements^27^. MR-compatible pressure force sensors were attached to the back and controlled for the pressure force of 30 Newton, and the pressure force was then transformed into an appropriate voltage signal^23^. The event-related fMRI experiment consisted of 17 stimuli of 5 s duration each, with a randomized inter-stimulus interval of 8–10 s for the L1 and the L4 stimulation level. The experimenter followed a shuffled stimulation protocol with an equal number of stimulations per vertebrae level, thus different for all participants. The stimulation level appeared onscreen in front of the experimenter in the MRI room, followed by a START signal to begin, and the applied force was displayed. A STOP signal indicated the end of the stimulation period.

#### Neuroimaging data acquisition

MRI data were acquired at the University Hospital of Zurich using a Philips Ingenia 3 T whole-body MR unit with a 32-channel head coil. Task-based functional scans consisting of 270 functional images (run of 540 s) involved a sensitivity-encoded single-shot (factor 41) T2*-weighted echoplanar imaging (EPI) sequence. Parameters were set as follows: repetition time [TR] = 2000 ms, echo time [TE] = 30 ms, field of view [FOV] = 220 mm × 220 mm, acquisition matrix size in plane = 72 × 74, interpolated to 128 × 128, 36 slices (slice thickness = 3 mm, inter-slice ga*P* = 0.6 mm) with a spatial resolution of 3 × 3 × 3 mm^3^ [reconstructed 1.72 × 1.72 × 3 mm^3^], flip angle = 78°, and sensitivity-encoded acceleration factor R = 1.8.

We placed the contiguous axial slices along the anterior–posterior commissural plane on a midsagittal scout image, covering the entire brain, including the cerebellum. The slices were acquired in ascending order. Three dummy scans were first acquired to reach steady-state magnetization and were subsequently discarded. We also acquired 3-dimensional T1-weighted anatomical images (160 slices; TR = 8.1 ms; TE = 3.7 ms; flip angle = 8°; FOV = 240 × 240 mm^2^; spatial resolution 1 × 1 × 1 mm^3^ [reconstructed 0.94 × 0.94 × 0.94 mm^3^]).

Imaging data analyses were conducted using SPM12 (Wellcome Department of Imaging Neuroscience, London, UK, http://www.fil.ion.ucl.ac.uk/spm) and CONN toolbox (version 18. a) software packages^69^ running on MATLAB R2019a (Mathworks Inc, Sherbon, MA). The preprocessing was performed with the SPM12 software and consisted of the following steps: realignment, slice timing correction, coregistration to structural T1-weighted image, segmentation, spatial normalization to Montreal Neurological Institute coordinates (MNI) space, and spatial smoothing (Gaussian kernel with full width at half maximum of 6 mm). Preprocessed data was imported into the CONN toolbox to perform the following processing steps: filtering (0.01 – 0.1 Hz) and denoising first-level and second-level analysis. Data from three participants showed excessive head motion during data acquisition (linear shift > 3 mm across the entire run and > 1.5 mm on a frame-to-frame basis, rotation > 1°). Head motions in any direction did not differ significantly between the two groups (t-test; t = 0.21, *P* < 0.8363).

The first-level analysis comprised two conditions, i.e., stimulation and no stimulation (rest) as the baseline. The stimulation condition was characterized by pressure on lumbar vertebrae (L1 and L4, respectively) with a duration of 5 s, and the absence of pressure defined the baseline. After convolving with the hemodynamic response function (implemented in SPM 12), a general linear model (GLM) with six movement parameters (3 translation and 3 rotation parameters) as a nuisance variable was applied for each subject’s data to yield the cortical activation during stimulation versus baseline. The statistical parametric maps of each subject were computed.

#### Group analysis of BOLD-fMRI activation

To test for group differences in fMRI activations (BOLD signal intensity) during stimulation *versus* rest, we performed a two-sample t-test using a random effect model. Statistical parametric maps were thresholded using a cluster-height threshold at *P* < 0.001 uncorrected at the voxel level and a cluster-extent threshold at *P* < 0.05 corrected for multiple comparisons applying a False Discovery Rate (FDR) correction.

#### Functional connectivity analysis

The event-related fMRI analysis was applied to test for differences in neural activation. Potentially detectable changes in the BOLD response reflect the changes in activity patterns in response to the stimulation, compared to the rest condition (without stimulation). While event-related fMRI analyses compare focal brain activity, functional connectivity investigates task-dependent connectivity strength between brain areas. To test whether patients and controls show significantly different task-modulation of functional connectivity between seed ROIs and all other brain voxels, a psycho-physiological interaction (PPI) analysis was applied. Preprocessed data was imported and post-processed to analyze functional connectivity using the CONN toolbox (version 18.a)^70^. The CONN Toolbox provides a generalized form of PPI (gPPI)^70^, simultaneously modeling the condition effects of all conditions and interactions in a single model. Cisler et al. have shown that gPPI analysis is more powerful than the standard PPI analysis^71^. The two main effects of tasks (i.e., stimulation and rest), the six motion parameters, and noise components from white matter and cerebrospinal areas were included in the model to regress out potentially confounding effects. Additionally, temporal band-pass filtering was applied (0.008 Hz < f < 0.09 Hz) to remove high-frequency fluctuations minimizing the impact of noise (e.g., physiological activity), and linear detrending was adopted. The adopted model for the first-level analysis comprised three variables: (1) A variable coding the two conditions (stimulation and rest) corresponding to the main psychological factor in the gPPI framework, (2) the seed ROI BOLD time-series (main physiological factor), and (3) the product of (1) and (2) representing the interaction term (i.e., the PPI term).

The investigated functional networks, chosen based on our a-priori hypotheses, were defined by the FSL Harvard–Oxford atlas as implemented in the CONN toolbox^69^: sensorimotor network with the seeds *lateral and superior sensorimotor network;* the salience network (SN) with the seeds *rostral prefrontal cortex*, *anterior insula, supramarginal gyrus (SMG),* and the *anterior cingulate cortex (ACC);* the limbic and anterior parahippocampal network comprising the seed *hippocampal gyrus, anterior division, hippocampus* and *amygdala* and finally, the anterior and posterior cerebellar networks. Based on a regression (bivariate) analysis, functional seed-to-voxel connectivity maps were created for each subject. Maps of regression coefficients associated with the interaction term were created and thresholded using a cluster-defining threshold of *P* < 0.001 (uncorrected) and an FDR-corrected cluster-level extent threshold of *P* < 0.05. Alternatively, the term “uncorrected” follows the *P*-value in the few cases when the activation did not survive correction for multiple comparisons but is still informative to describe. The coordinates in MNI space, cluster size, z-value, and *P*-value are reported for each peak activation. For labeling the anatomical localization of the significant activation peaks, we used the terminology from the Harvard–Oxford Atlas.

## Supplementary Information

Supplementary Information.

## Data Availability

The datasets used and/or analyzed during the current study are available from the corresponding author upon reasonable request.
