# Supplementary material for: Adaptive changes in sensorimotor processing in patients with acute low back pain
Source: Sci Rep. 2022 Dec 16;12:21741. doi: 10.1038/s41598-022-26174-2 (PMC9758154; doi:10.1038/s41598-022-26174-2)
Supplement: Supplementary file 1 — Supplementary Information. [file 41598_2022_26174_MOESM1_ESM.docx]

**Supplementary Material Table 1**

**Adaptive Changes in Sensorimotor Processing in Patients with Acute Low Back Pain**

Bart Boendermaker^1,2,4^, Roman Buechler^2^, Lars Michels^2,3^, Jo Nijs^4^, Iris Coppieters^4^, Sabina Hotz-Boendermaker^1*^

**Table 2.** Event-related activations due to non-painful posterior to anterior intervertebral movement (PA) onto the lumbar spinous processes L1 and L4 (corrected for multiple comparisons applying a False Discovery Rate (FDR) correction)

| **Control group** | |  |  | **MNI peak coordinates** | | |  |  |  |
| --- | --- | --- | --- | --- | --- | --- | --- | --- | --- |
| **Voxel** | **p(FDR)** | **Z** |  | **x** | **y** | **z** | **Brain regions** | | **Functional region** |
| **282** | **0.036** | **4.24** | **R** | **54** | **-28** | **18** | Parietal Opercular Cortex | | S2 |
|  |  |  |  | **66** | **-24** | **20** | Supramarginal Gyrus | | S2 |
| **482** | **0.005** | **4.13** | **L** | **-50** | **-32** | **20** | Parietal Opercular Cortex | | S2 |
|  |  |  |  | -62 | -40 | 36 | Supramarginal Gyrus | |  |
| 108 | 0.247 | 4.75 | L | -52 | -2 | 4 | Central Opercular Cortex | | S2 |
| 161 | 0.172 | 4.75 | R | 18 | -36 | 72 | Postcentral Gyrus | | S1 |
| 147 | 0.172 | 4.65 | L | -60 | -60 | 4 | Middle Temporal Gyrus | |  |
| 124 | 0.214 | 4.65 | L | -18 | -34 | 72 | Postcentral Gyrus/Supramarginal Gyrus | | S1 |
| 42 | 0.862 | 4.06 | R | 4 | 0 | 64 | Juxtapositional Lobule | | SMA |
| 40 | 0.862 | 4.06 | R | 12 | -34 | 54 | Postcentral Gyrus | |  |
|  |  |  |  |  |  |  |  |  |  |
|  |  |  |  |  |  |  |  |  |  |
| **Patient group** | |  |  | **MNI peak coordinates** | | |  |  |  |
| **Voxel** | **p(FDR)** | **Z** |  | **x** | **y** | **z** | **Brain regions** | | **Functional region** |
| **3443** | **0.001** | **5.17** | **R** | **46** | **-30** | **26** | Parietal Opercular Cortex | | S2 |
|  |  | **5.15** |  | **58** | **-18** | **20** | Central Opercular Cortex | |  |
| **588** | **0.001** | **4.87** | **L** | **-64** | **-24** | **22** | Supramarginal Gyrus, anterior division | | |
|  |  | **4.48** |  | **-56** | **-28** | **18** | Parietal Opercular Cortex | | |
| **413** | **0.001** | **4.78** | **L** | **-60** | **8** | **8** | Precentral Gyrus | | S2 |
|  |  | **3.93** |  | **-42** | **-4** | **6** | Insular Cortex | |  |
|  |  | **3.85** |  | **-56** | **-2** | **6** | Central Opercular Cortex | |  |
| **125** | **0.05** | **4.67** | **R** | **22** | **-32** | **70** | Postcentral Gyrus | | S1 |
| **328** | **0.001** | **4.3** | **R** | **2** | **-30** | **54** | Precentral Gyrus/Postcentral Gyrus | | |
| **162** | **0.021** | **4.11** | **L** | **-4** | **4** | **42** | Cingulate Gyrus, anterior division | | |
| 92 | 0.086 | 4.32 | R | 4 | -92 | 16 | Occipital Pole | |  |
|  |  | 3.79 | L | -2 | -14 | 48 | Juxtapositional Lobule | | SMA |
| 103 | 0.071 | 3.97 | L | -18 | -34 | 64 | Postcentral Gyrus | | S1 |
| 71 | 0.133 | 3.94 | L | -10 | 38 | 30 | Paracingulate Gyrus | |  |
| 75 | 0.129 | 3.81 | R | 4 | -6 | 64 | Juxtapositional Lobule | | SMA |
| 54 | 0.215 | 3.76 | R | 22 | -100 | -2 | Occipital Pole | |  |
| 32 | 0.464 | 3.71 | R | 50 | -40 | 10 | Supramarginal Gyrus, posterior division | | |

In bold significant cluster, L = left; R = right; S1, primary somatosensory cortex; S2, secondary somatosensory cortex; SMA, supplementary motor area
